# Sensing Molecules with Metal–Organic Framework Functionalized Graphene Transistors

**DOI:** 10.1002/adma.202103316

**Published:** 2021-09-08

**Authors:** Sandeep Kumar, Yohanes Pramudya, Kai Müller, Abhinav Chandresh, Simone Dehm, Shahriar Heidrich, Artem Fediai, Devang Parmar, Delwin Perera, Manuel Rommel, Lars Heinke, Wolfgang Wenzel, Christof Wöll, Ralph Krupke

**Affiliations:** ^1^ Institute of Nanotechnology Karlsruhe Institute of Technology 76021 Karlsruhe Germany; ^2^ Institute of Materials Science Technische Universität Darmstadt 64287 Darmstadt Germany; ^3^ Institute of Functional Interfaces Karlsruhe Institute of Technology 76021 Karlsruhe Germany; ^4^ Institute of Quantum Materials and Technologies Karlsruhe Institute of Technology 76021 Karlsruhe Germany

**Keywords:** alcohol, graphene, metal–organic frameworks, sensing, transistors

## Abstract

Graphene is inherently sensitive to vicinal dielectrics and local charge distributions, a property that can be probed by the position of the Dirac point in graphene field‐effect transistors. Exploiting this as a useful sensing principle requires selectivity; however, graphene itself exhibits no molecule‐specific interaction. Complementarily, metal–organic frameworks can be tailored to selective adsorption of specific molecular species. Here, a selective ethanol sensor is demonstrated by growing a surface‐mounted metal–organic framework (SURMOF) directly onto graphene field‐effect transistors (GFETs). Unprecedented shifts of the Dirac point, as large as 15 V, are observed when the SURMOF/GFET is exposed to ethanol, while a vanishingly small response is observed for isopropanol, methanol, and other constituents of the air, including water. The synthesis and conditioning of the hybrid materials sensor with its functional characteristics are described and a model is proposed to explain the origin, magnitude, and direction of the Dirac point voltage shift. Tailoring multiple SURMOFs to adsorb specific gases on an array of such devices thus generates a versatile, selective, and highly sensitive platform for sensing applications.

## Graphical abstract

A novel concept of sensing molecules is introduced, which combines the sensitivity of a graphene field‐effect transistor with the selectivity of a metal–organic framework. As a prototype example, an ethanol sensor is fabricated that is insensitive to other alcohols and humidity. The readout signal is a shift in the Dirac voltage.

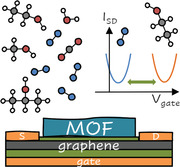

## 1 Introduction

2D materials in the form of monolayers constitute atomically thin and extended planar structures with inherently entangled “bulk” and “surface” properties. As such, 2D materials are highly susceptible to electric fields induced by gate voltages and local charge distributions,^[^ ^1^, ^2^, ^3^, ^4^ ^]^ physisorption, chemisorption, interfacial doping,^[^ ^5^, ^6^, ^7^, ^8^ ^]^ light, stress, and strain.^[^ ^9^, ^10^, ^11^, ^12^ ^]^ Consequently, 2D materials are of great interest in a range of sensing applications, as has been demonstrated for the detection of gases,^[^ ^13^, ^14^, ^15^ ^]^ metal ions,^[^ ^16^, ^17^ ^]^ strain,^[^ ^9^ ^]^ and pressure.^[^ ^18^ ^]^ A challenge for the field is the limited availability of large‐area 2D materials. Currently, graphene is the only commercially available wafer‐scale material with well‐defined properties. Graphene has been demonstrated to be highly sensitive to interfacial doping, electric fields, and vicinal dielectrics, and for a reliable and easily accessible readout the position of the Dirac point in the gate‐voltage dependence of a graphene field‐effect transistor (GFET) can be exploited. This sensing principle has already been demonstrated for pH measurements,^[^ ^19^ ^]^ where a dielectric oxide provides the sensitivity toward hydronium ions. However, for general applicability, an interfacial layer is required that can be tailored to be highly selective to specific molecules, offering a larger versatility as compared to surface functionalization. Metal–organic frameworks (MOFs) are a class of porous materials and a selective absorptivity for specific molecules can be tailored by specific combinations of metal ions or clusters and organic ligands. Countless MOFs have been synthesized and exploited for many applications related to energy harvesting^[^ ^20^ ^]^ and gas storage.^[^ ^21^, ^22^, ^23^ ^]^ Fewer examples of MOF‐based sensors exist for gas sensing,^[^ ^24^, ^25^ ^]^ chemical sensing,^[^ ^26^ ^]^ and small molecule detection. These devices exploit the induced stress in the MOF crystal structure,^[^ ^27^ ^]^ and/or changes in the luminescence of the MOF in the presence of metal ions.^[^ ^28^ ^]^ Recently, a MOF with high affinity to SO_2_ was grown between interdigitated electrodes, and the change in the capacitance of the structure under gas uptake was used for SO_2_ detection.^[^ ^29^ ^]^

Here, we demonstrate a selective sensing platform by growing a surface‐mounted metal–organic framework (SURMOF) directly onto a GFET. We hypothesized that such a device can benefit from the high sensitivity, easy readout capability of a GFET, and the high selectivity of a SURMOF. We present high mobility SURMOF/GFETs sensors based on Cu_2_(BDC)_2_SURMOF‐2, which yield a selective sensitivity to ethanol and show that the SURMOF substantially changes the response of the GFET against specific gas molecules in the air. We determine the sensitivity and response time of the ethanol sensor, and describe the activation and resetting schemes. To support these findings, we correlate the electrical transport characteristics with Raman spectroscopy, provide in addition energy‐dispersive X‐ray (EDX), X‐ray diffraction (XRD), scanning electron microscopy (SEM), adsorption and desorption data, and propose a model for the response of the sensor.

## 2 Results and Discussion

**Figure 1:**
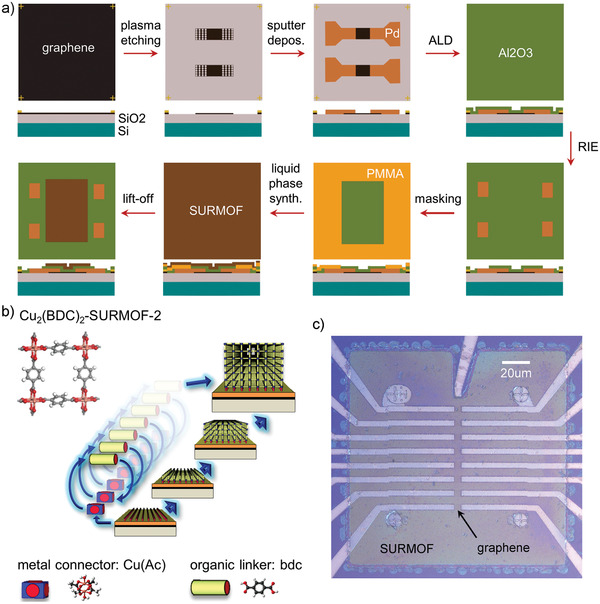
Fabrication of SURMOF/GFET devices. a) Process flow involving multiple electron beam lithography patterning, etching, and deposition steps as described in the main text and the Experimental Section. b) Liquid‐phase synthesis of the SURMOF on the GFET by repeated exposure to metal connectors and organic linkers. c) Optical microscopy image of the final SURMOF/GFET devices. The sensor areas are located between the metal source–drain electrodes where graphene can be recognized due to the optical transparency of the SURMOF.

The SURMOF/GFET sensors were fabricated by employing several steps of electron‐beam lithography, metallization, etching, coating, atomic layer deposition (ALD), and liquid‐phase synthesis, as shown in the schematic process flow in **Figure** **1**a. Large‐area monolayer chemical‐vapor‐deposition (CVD)‐grown graphene on p^++^Si/300‐nm‐SiO_2_ was etched by plasma oxidation into multiple 5 µm wide and 100 µm long strips, each electrically contacted separately with Pd/Cr source–drain electrodes. The uncovered graphene channels have a length of 5 µm with an active area of 25 µm^2^. To minimize the contact resistance, the graphene underneath the electrodes was perforated with holes.^[^ ^30^ ^]^ 5 nm of Al_2_O_3_ was grown by thermal ALD by subsequent pulses of trimethylaluminum (TMA) and ozone^[^ ^31^ ^]^ over the entire structure to provide surface hydroxyl groups required for the SURMOF growth and to electrically isolate the graphene and the electrodes. The contact pads were opened by local etching and the area for the SURMOF growth was defined by a resist mask. A Cu_2_(BDC)_2_‐SURMOF‐2 with a nominal thickness of 100 nm was grown by liquid‐phase synthesis (Figure 1b),^[^ ^32^, ^33^ ^]^ using a spray synthesis similar to Hurrle et al.^[^ ^34^ ^]^ Afterward, the SURMOF was lifted‐off from outer areas with a resist mask. An optical microscopy image of the complete SURMOF/GFET devices is shown in Figure 1c. We found that the Al_2_O_3_ layer is required for the SURMOF to grow on top of graphene. With energy‐dispersive X‐ray spectroscopy, we have verified that the ALD‐grown Al_2_O_3_ layer is still intact after the SURMOF synthesis (Figure S1, Supporting Information). Out‐of‐plane XRD measurements on a large‐area reference sample show that the SURMOF grows on 5 nm Al_2_O_3_/graphene/300‐nm‐SiO_2_/Si with the (001) orientation parallel to the substrate surface and with a lattice constant of 1.12 nm (Figure S2, Supporting Information), which is comparable to the synthesis of the SURMOF on other —OH terminated surfaces.^[^ ^33^, ^35^ ^]^ Despite the flat appearance of the SURMOF in the optical image and the (001) reflex in the XRD data, the SURMOF layer is rough on a microscopic scale. SEM images recorded under different angles reveal that the SURMOF at the interface with the Al_2_O_3_ layer is continuous, whereas the upper surface is uneven, with small SURMOF crystals pointing in various directions (Figure S3, Supporting Information), likely a consequence of the atomically uneven Al_2_O_3_ surface.

After fabrication, the devices were wired to a ceramic package, mounted to a cavity of volume ≈1 cm^3^, and connected to a gas line system, described by Ganzhorn et al.^[^ ^36^ ^]^ A four‐way valve enables instantaneous switching between gases, and the conditions were controlled by pressure gauges and flow meters (dosing valves). Devices were exposed to air, N_2_, O_2_, and CO_2_, and to molecules from liquids by purging N_2_ through H_2_O, methanol (CH_3_OH), ethanol (C_2_H_5_OH), and isopropanol (C_3_H_7_OH). The relative humidity and alcohol concentrations were monitored downstream with commercial sensors. The gate‐voltage dependencies of the device conductance were measured with a semiconductor parameter analyzer at 300 K, and the doped silicon was used as a back‐gate for all devices. More details in the Experimental Section.

**Figure 2:**
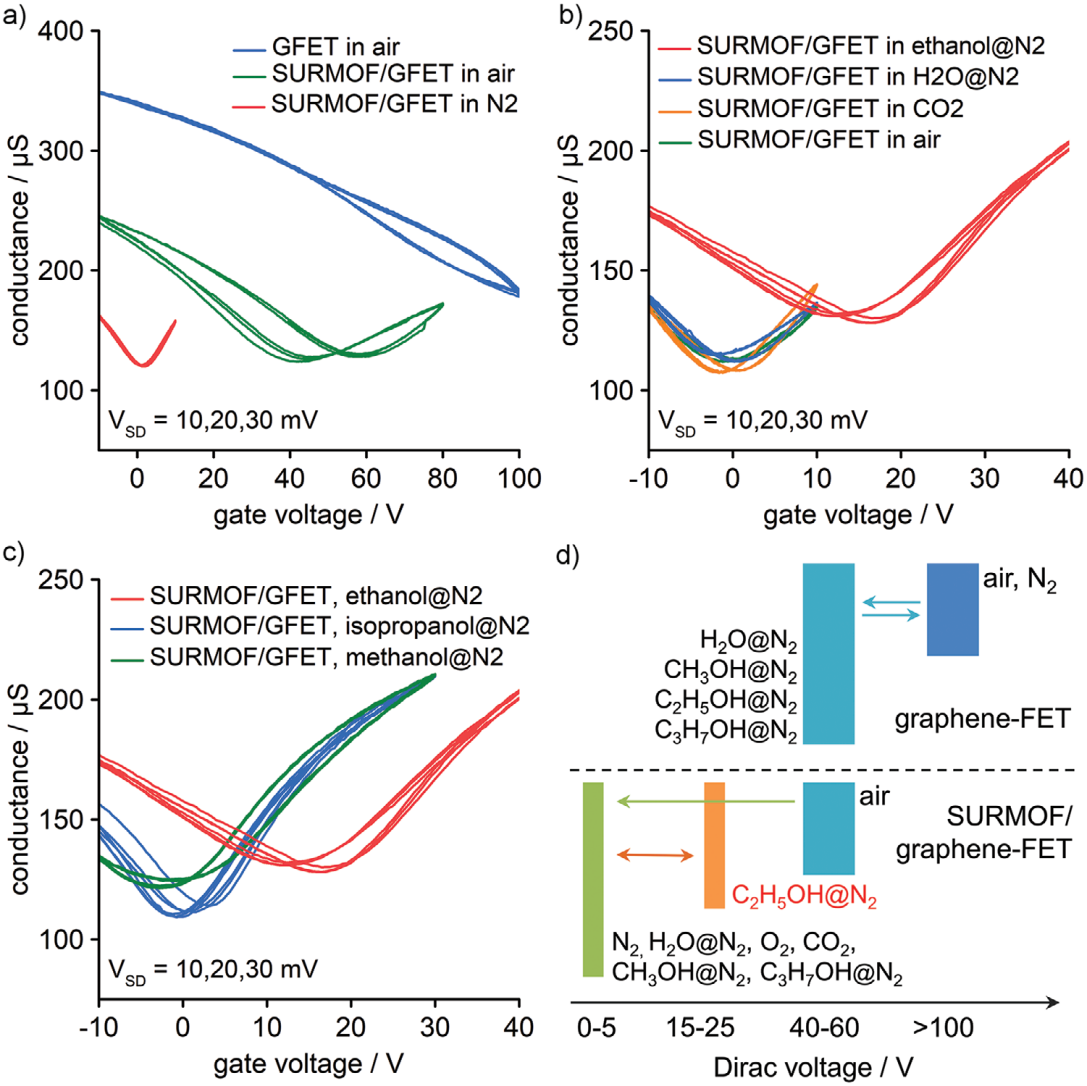
Gate‐voltage dependence of SURMOF/GFET device conductance. a) Response to air before (GFET in air) and after SURMOF coating (SURMOF/GFET in air), and to N_2_ after SURMOF activation (SURMOF/GFET in N_2_). b) Response of SURMOF/GFETs to air and CO_2_, and N_2_ purged with H_2_O and ethanol. c) Response of SURMOF/GFETs to N_2_ purged with ethanol, methanol, and isopropanol. d) Schematic overview on the responses of the SURMOF/GFET and GFET devices and the Dirac voltages measured under the indicated conditions.

First, we discuss the gate‐voltage dependence of the conductance of GFETs without SURMOF coating. As a measure for the doping, we use the Dirac voltage, which is defined as the gate voltage that yields the minimum conductance. If the applied gate voltage is equal to the Dirac voltage, the Fermi level coincides with the charge neutrality point of graphene at the K point of the graphene band structure. The devices show significant hole‐doping in air, but also while purging with dry N_2_, as shown by the blue traces in **Figure** **2**a and Figure S4 in the Supporting Information, respectively. The conductance decreases monotonically with increasing gate voltage and the Dirac voltage *V*_Dirac_ is beyond the maximum applied gate voltage of +100 V. We can calculate the corresponding doping level from the carrier concentration *n* in the graphene layer, which depends on gate voltage *V*_gate_, the Dirac voltage *V*_Dirac_, the geometrical capacitance *C* formed between graphene and SiO_2_/Si, and the graphene quantum capacitance, using *n*(*V*_gate_,*V*_Dirac_) =−sign(*V*_gate_ − *V*_Dirac_)(sqrt(|*V*_gate_ − *V*_Dirac_|/*a* + *c* ^2^) − *c*)^2^,^[^ ^37^ ^]^ with *c* = *b*/(2*a*), *a* = *e*/*C*, *b* = *h* *v*_F_π^0.5^/*e*, *v*_F_ = 10^6^ m s^−1^, and *C* = 11.5 nF cm^−2^ for our devices.

**Figure 3:**
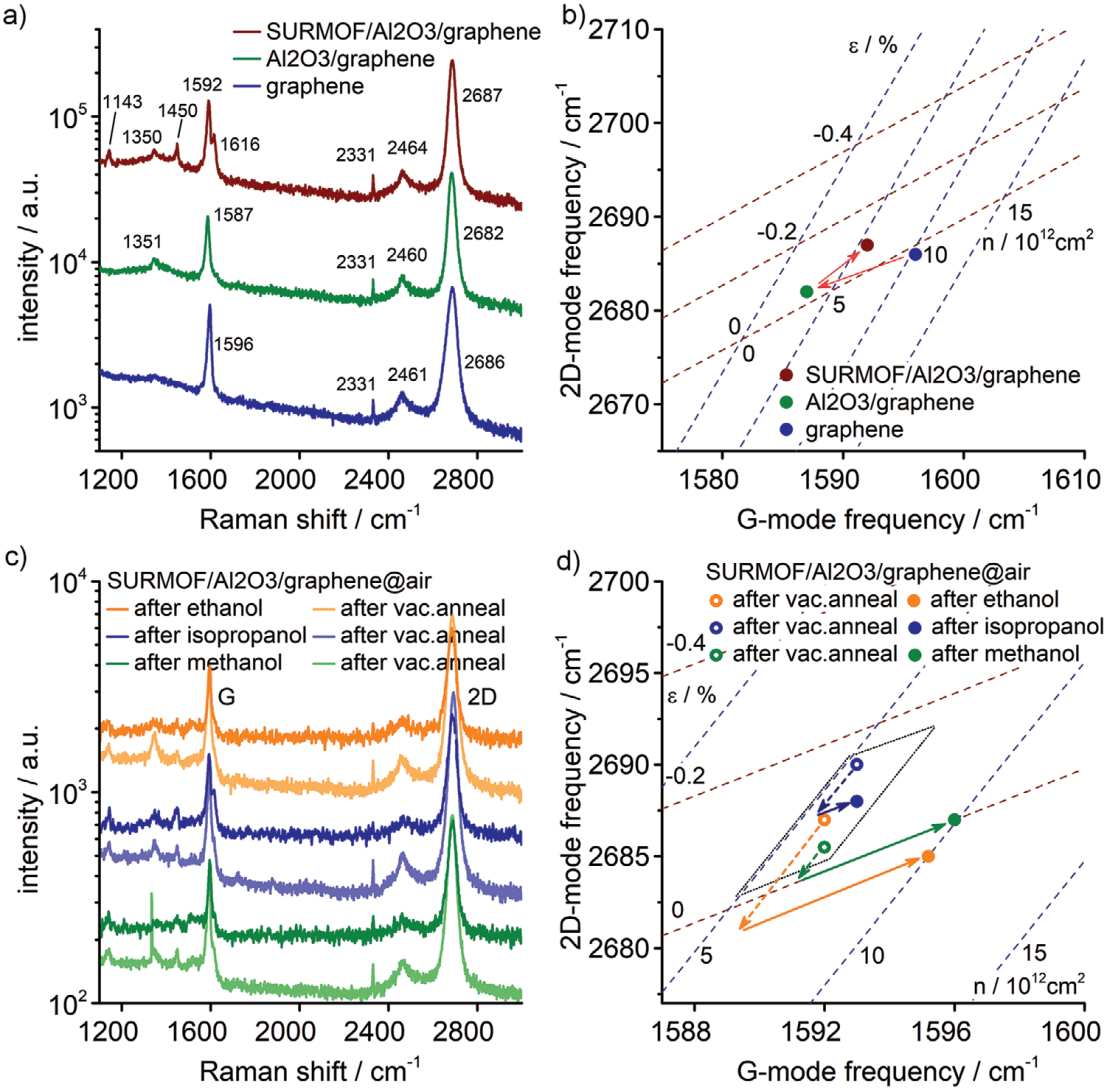
a) Raman spectra of a CVD‐graphene/300‐nm‐SiO_2_/Si sample before and after Al_2_O_3_ and SURMOF growth measured in air. The peak at 2331 cm^−1^ is from N_2_(g). The upper two curves are shifted for clarity. b) Correlation between the frequencies of the G and 2D Raman modes shown in (a). The dashed lines show the effects of hole‐doping (Δω_2D_/Δω_G_ = 0.7) and strain (Δω_2D_/Δω_G_ = 2.2) on the mode frequencies of graphene using the model of Lee et al.^[^ ^38^ ^]^ c) SURMOF/5‐nm‐Al_2_O_3_/graphene/300‐nm‐SiO_2_/Si reference sample measured in air after exposure to ethanol, isopropanol, and methanol vapor. Before alcohol vapor exposure, the samples were vacuum annealed at 150 °C. All curves except the lowest are shifted for clarity. d) Correlation between the frequencies of the G and 2D Raman modes shown in (c). The dashed and full arrows show the corresponding changes in strain and doping. The dotted box encompasses the data of the vacuum annealed samples and defines the error for determining the relative changes in strain and doping.

The carrier concentration at zero gate voltage due to doping is then given as *n*_doping_ = *n*(*V*_gate_ = 0,*V*_Dirac_), and we obtain *n*_doping_ > +7 × 10^12^ cm^−2^ for *V*_Dirac_ > +100 V; *V*_Dirac_ = +150 V would yield *n*_doping_ = 10^13^ cm^−2^. The doping level of the graphene layer was also followed by Raman spectroscopy. The spectrum of a reference CVD‐graphene/300‐nm‐SiO_2_/Si sample in **Figure** **3**a shows the graphene G‐peak at 1596 cm^−^^1^, the 2D‐peak at 2686 cm^−1^, and no visible D‐peak. In Figure 3b, the peak positions were analyzed with the model of Lee et al.,^[^ ^38^ ^]^ by plotting our data onto the dashed grid lines, which shows how the G‐peak and 2D‐peak positions depend on doping and strain. Data points can then be deconvoluted into components of strain and doping by projection onto the grid lines, as demonstrated in Figure 3d. The data is in Figure 3a,b consistent with a hole‐doped graphene layer, which is nearly strain‐ and defect‐free. The Raman peak in Figure 3a at 2461 cm^−1^ is from a two‐phonon combination mode in graphene, referred to as G* or D+D″.^[^ ^39^ ^]^ The peak at 2331 cm^−1^ originates from ambient molecular nitrogen above the sample surface.^[^ ^40^, ^41^ ^]^ After the growth of a nominally 5 nm thick Al_2_O_3_ layer, a small D peak appears in Figure 3a at 1351 cm^−1^, and the G‐ and 2D‐peaks are shifted down to 1589 and 2682 cm^−1^, respectively. It indicates that the growth of Al_2_O_3_ induces a moderate defect concentration in the graphene layer and it reduces slightly the hole‐doping as shown in Figure 3b. The graphene layer though remains free of strain. Note that the Raman measurement conditions (ambient, laser irradiation) are not strictly identical with the GFET characterization conditions (gas flow system, dark), but the data is in agreement with the transport data and shows that the graphene layer underneath the Al_2_O_3_ layer is hole‐doped.

We have first explored the sensitivity of the GFET without SURMOF to the presence of alcohol and water vapors. Figure S4a–c in the Supporting Information shows the response of the GFET to methanol‐, ethanol‐, and isopropanol molecules in the N_2_ stream. We observe that the overall conductance is reduced in the presence of all three alcohols and the corresponding Dirac voltages could be determined. The amount of hole‐doping reduction is similar but most pronounced during exposure to methanol (*n*_doping_ = 3.3–3.5 × 10^12^ cm^−2^), followed by ethanol (*n*_doping_ = 4.6–5.7 × 10^12^ cm^−2^), and isopropanol (*n*_doping_ = 4.3–6.0 × 10^12^ cm^−2^). Also, the exposure to water molecules reduces the hole‐doping when the gas flow is changed from dry N_2_ to humid N_2_ (≈80% relative humidity; Figure S4d, Supporting Information). All changes are reversible and strong hole‐doping is re‐established when purging the devices with dry N_2_ (Figure S4, Supporting Information). It is important to note that GFETs without SURMOF coating always remain in the strong hole‐doping regime, with the Dirac voltage above 40 V, independent of the type of alcohol exposure. This is summarized schematically in the upper part of Figure 2d. The growth of the SURMOF changes this behavior completely. The Dirac voltage of the SURMOF/GFET in air is at 40–60 V, whereas without SURMOF, it was beyond 100 V, as shown in Figure 2a. Also, the Raman spectrum has changed in Figure 3a. After the growth of a nominally 100 nm thick SURMOF layer, the G and 2D‐peaks shifted upward to 1592 and 2687 cm^−1^, respectively. The shifts in Figure 3b indicate that the SURMOF growth introduces about 0.1% compressive strain in the graphene layer and a slight change in doping. After the SURMOF growth, three additional Raman peaks are observed at 1143, 1450, and 1616 cm^−1^, assigned to the ring stretch of benzene‐dicarboxylate, asymmetric CO stretch, and C=C stretch in the SURMOF layer, respectively.^[^ ^42^ ^]^ Again, absolute strain and doping values should be taken with care.

Most interestingly, the hole‐doping in air is not only significantly reduced for GFETs after SURMOF growth, but after purging with N_2_, the device becomes undoped, and the Dirac voltage approaches zero, as shown in Figure 2a. Also, the mobility has increased to 4640 cm^2^ V^−1^ s^−1^, which is remarkably high for CVD‐graphene on SiO_2_/Si without vacuum or current annealing.

The purging of the SURMOF with N_2_ is a common activation process step,^[^ ^43^ ^]^ promoting the desorption of solvent and guest molecules from MOF pores. The details of the desorption process can be rather complex. For instance, the desorption of water is inhibited in the presence of ethanol due to complex formation at the outer surface of an MOF.^[^ ^42^ ^]^ The activation process is important also in this work and essentially constitutes the last step in the SURMOF synthesis. The outcome of this activation process implies that ethanol captured in the SURMOF from the synthesis process diffuses out while purging with N_2_, revealing an undoped SURMOF/GFET. It is expected that ethanol is able to diffuse back into the structure as well.

We have exposed the SURMOF/GFET to a flow of N_2_ saturated with ethanol (ethanol@N_2_). Indeed, Figure 2b shows that the Dirac voltage shifts from 0 to 15 V when switching from N_2_ to ethanol@N_2_. The data also show that the SURMOF/GFET is insensitive to H_2_O molecules (H_2_O@N_2_), CO_2_, and air. Furthermore, Figure 2c shows that the Dirac voltage of the SURMOF/GFET remains at zero and is not reacting to N_2_ saturated with methanol (methanol@N_2_) or isopropanol (isopropanol@N_2_). These results are summarized schematically in the lower part of Figure 2d.

**Figure 4:**
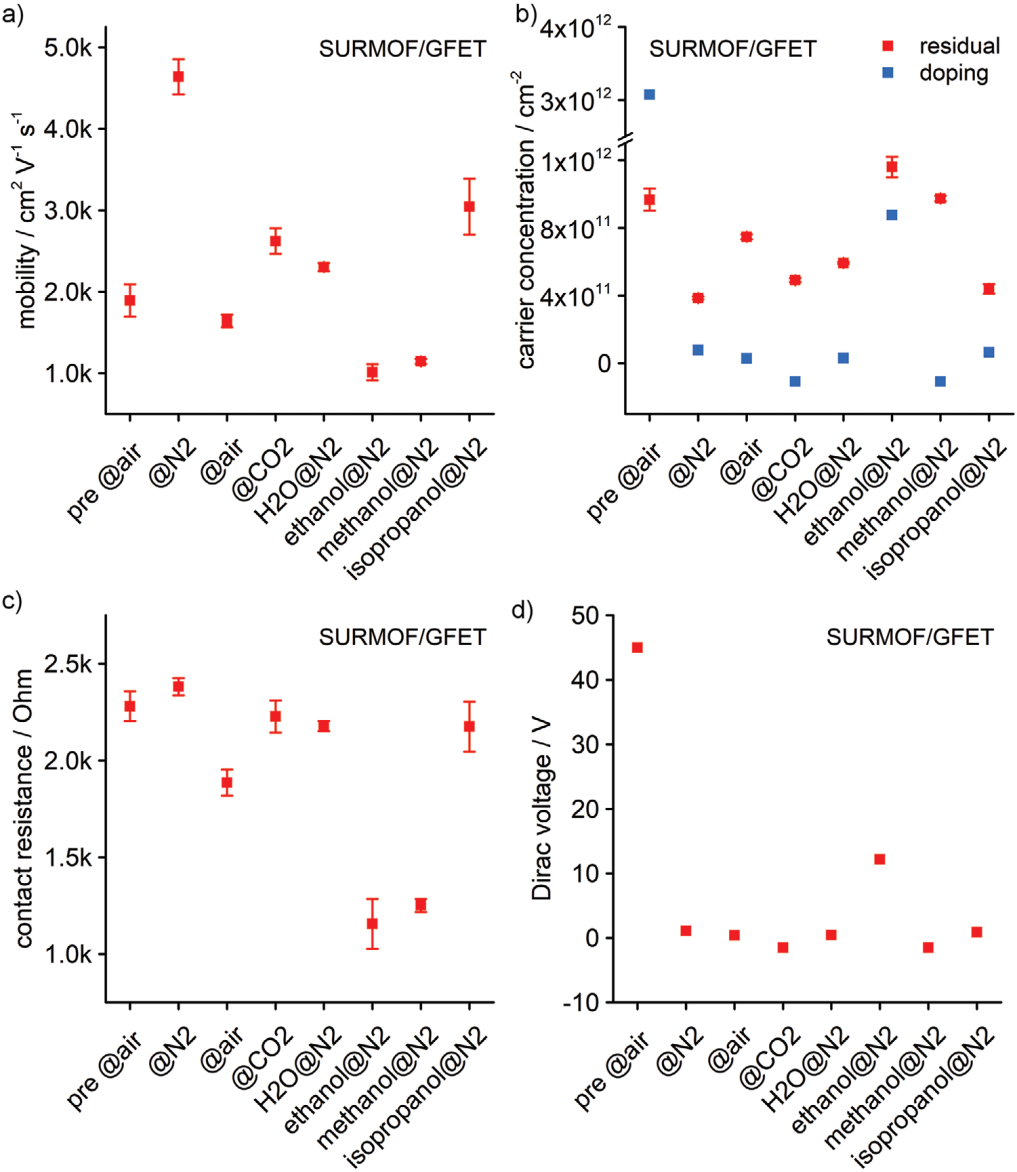
a–d) Comparison of mobility (a), residual carrier concentration and doping (b), contact resistance (c), and Dirac voltage (d) of SURMOF/GFET devices under various conditions. The values have been determined by fitting the transport measurements to the model of Kim et al.^[^ ^37^ ^]^ The carrier concentration due to doping in (b) has been derived from the Dirac voltage. All data acquired after SURMOF activation, except for the measurement in air before activation (pre @air).

The selectivity of the Dirac voltage to ethanol likely results from changes in the SURMOF/Al_2_O_3_ interface since the adsorption and desorption properties of the SURMOF alone are very similar for the three alcohols (Figure S5, Supporting Information). More insights were gained by analyzing the SURMOF/GFETs transconductance curves with the model of Kim et al.^[^ ^37^ ^]^ We determined for all conditions the mobility, residual carrier concentration, contact resistance, and Dirac voltage. **Figure** **4**a shows that the highest mobility has been measured in pure nitrogen (≈4640 cm^2^ V^−1^ s^−1^) after the activation procedure. The lowest mobility is obtained for ethanol@N_2_ (1020 cm^2^ V^−1^ s^−1^) and methanol@N_2_ (1150 cm^2^ V^−1^ s^−1^). The mobility for isopropanol@N_2_ is 3050 cm^2^ V^−1^ s^−1^, and thereby significantly higher than for the other two alcohols. The mobility for all other conditions is between ≈1600 and 2600 cm^2^ V^−1^ s^−1^. Also, the residual carrier concentration in Figure 4b follows a similar pattern. The lowest value is observed in pure nitrogen (3.8 × 10^11^ cm^−2^), the highest values for ethanol@N_2_ (1.2 × 10^12^ cm^−2^) and methanol@N_2_ (9.7 × 10^11^ cm^−2^), comparable to air@N_2_ (9.7 × 10^11^ cm^−2^) before the activation procedure. The value for isopropanol@N_2_ is 4.4 × 10^11^ cm^−2^, and thereby significantly lower than for the other two alcohols. The other values are in between (4.9–7.5 × 10^11^ cm^−^^2^). Furthermore, the contact resistance in Figure 4c reproduces the different behavior of the alcohols. The lowest contact resistance is observed for ethanol@N_2_ (1156 Ohm) and methanol@N_2_ (1251 Ohm). For isopropanol@N_2_, the value is 2175 Ohm, and thereby again significantly different from the other two alcohols. The contact resistance for all other conditions is between 1.9 and 2.3 kOhm. On the other hand, the Dirac voltage in Figure 4d and the corresponding doping level in Figure 4b show a different behavior among the exposure to the alcohol molecules. For ethanol@N_2_, the Dirac voltage is at 12 V and the doping at 8.8 × 10^11^ cm^−2^, whereas both values are significantly smaller for methanol@N_2_ (1.5 V, 1.07 × 10^11^ cm^−^^2^) and isopropanol@N_2_ (+0.9 V, 6.4 × 10^10^ cm^−2^). The data thus leads us to the conclusion that ethanol and methanol molecules reach the SURMOF/Al_2_O_3_ interface but isopropanol does not, and that only ethanol molecules induce a shift in the Dirac voltage. We can conclude that the MOF/GFET sensing of ethanol is due to the shift in the Dirac voltage and not due to changes in the carrier mobility, as can be seen by the comparison between the corresponding ethanol and methanol data in Figure 4a,d, which shows similar mobility for both alcohols but very different Dirac voltages. This result may not be surprising given that the graphene layer is ≈3–5 nm below the Al_2_O_3_ surface making the charge carriers in graphene less susceptible to scattering by molecules. Also, Schedin et al. reported no change in mobility despite exposing graphene directly to various gases.^[^ ^44^ ^]^

Further insight into the alcohol‐induced doping of graphene was gained by Raman spectroscopy on a reference SURMOF/5‐nm‐Al_2_O_3_/graphene/300‐nm‐SiO_2_/Si sample. Figure 3c shows changes to the Raman spectra when exposed to ethanol, isopropanol, and methanol vapors. Additional measurements were taken immediately after vacuum annealing at 150 °C, before each alcohol exposure. Once again we used the correlation between the G and 2D peak positions to determine the doping and strain in the graphene layer.^[^ ^38^ ^]^ No effect is observed after exposure to isopropanol. The changes in strain and doping are within uncertainty, as shown in Figure 3d. This result supports the transport data, which indicates that isopropanol does not reach the SURMOF/Al_2_O_3_ interface. Also in agreement with the transport data is the effect due to exposure to ethanol molecules. The Raman data shows that ethanol does reach the interface and induces significant hole‐doping in the graphene layer. Regarding the exposure to methanol, both Raman and transport data show that methanol reaches the SURMOF/Al_2_O_3_ interface. However, the transport data shows no change in doping, whereas the Raman data does. It is important to note that Raman probes the local doping and that a shift of the G peak to larger wavenumbers occurs for electron‐doping and hole‐doping.^[^ ^45^, ^46^ ^]^ Transport measurements instead probe the average net charging by a shift of the Dirac voltage. The formation of electron–hole puddles with zero net charges, as reported in the absence of charged impurities,^[^ ^47^ ^]^ could explain the observation with methanol, and their presence would reduce the carrier mobility and increase the residual carrier concentration without shifting the Dirac voltage. At the same time, a shift of the Raman G‐peak frequency should be observable because of local doping. The different interactions of ethanol and methanol with the SURMOF/Al_2_O_3_ interface though must have a microscopic origin and Figure 3d indicates a small difference in the change of strain in the graphene layer.

**Figure 5:**
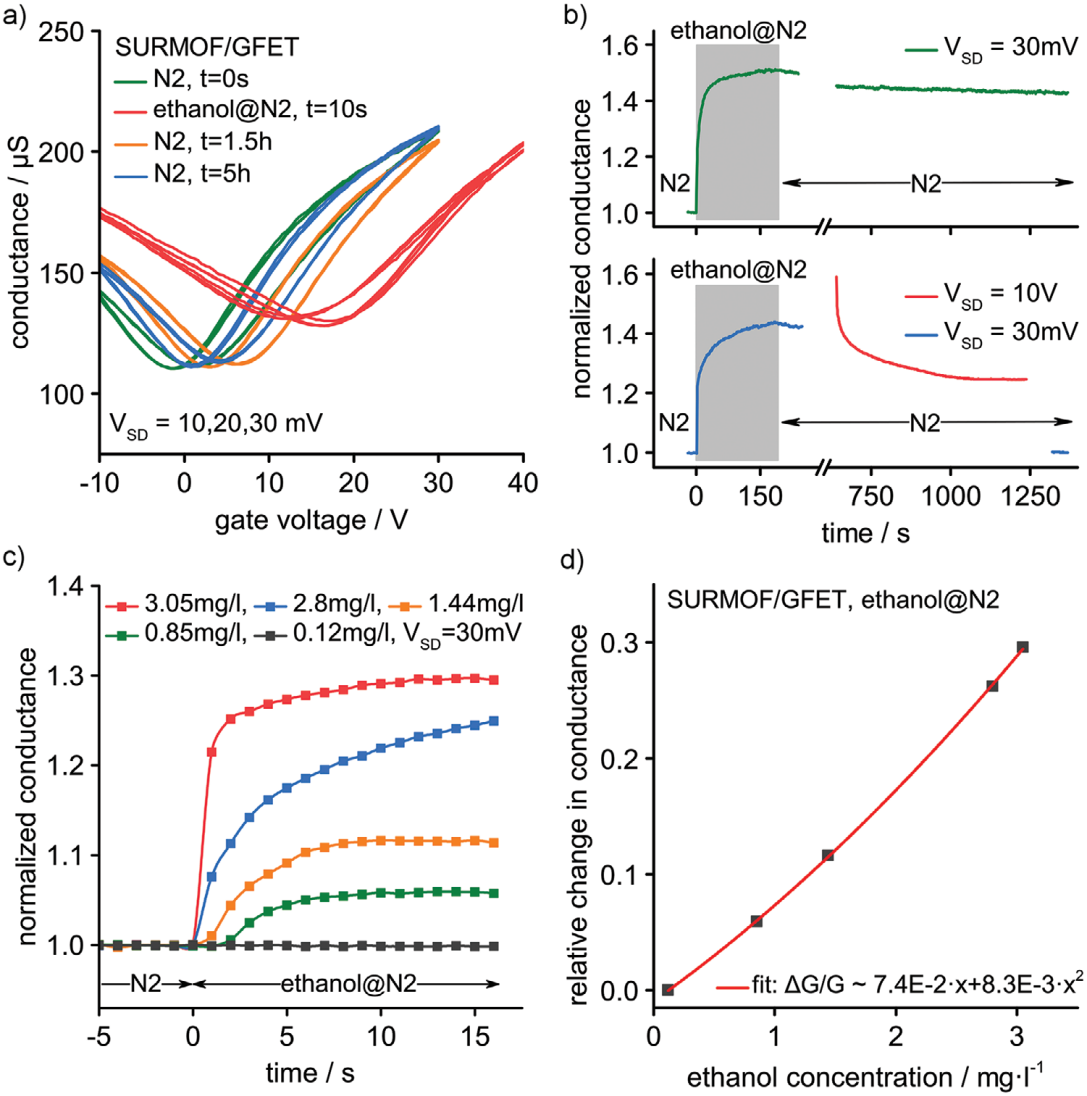
SURMOF/GFET time response and sensitivity to exposure of ethanol. a) Time evolution of conductance versus gate voltage measured before, after, and during ethanol exposure. Time steps and source–drain voltages *V*_SD_ are given. b) Top: the corresponding change in conductance was measured at zero gate voltage and *V*_SD_ = 30 mV (green trace). Bottom: The reset procedure with a temporary increase of *V*_SD_ from 30 mV (blue trace) to 10 V (red trace). After the reset, the conductance reaches the initial value (see blue trace for *t* > 1250 s @*V*_SD_ = 30 mV). c) SURMOF/GFET response versus ethanol concentration. The concentrations were measured with a commercial ethanol sensor. d) Change in conductance after 15 s of ethanol exposure versus ethanol concentration. The data are fitted with a second‐degree polynomial function.

To further explore the sensor performance, we investigated the response time and sensitivity of the SURMOF/GFET devices. **Figure** **5**a shows that the Dirac voltage changes from 0 to 15 V within seconds when switching from N_2_ to ethanol@N_2_. However, after switching back to pure N_2_, it takes several hours until the Dirac voltage reapproaches 0 V. In the upper part of Figure 5b, we have continuously monitored the adsorption and desorption of ethanol by measuring the conductance at a fixed gate voltage of 0 V and a source–drain voltage of 30 mV. After 20 min of purging with N_2_, the conductance is still 40% above the value before ethanol@N_2_ exposure. Such slow desorption is a serious obstacle for sensing applications. Fortunately, there is a simple way to overcome the problem by thermally activating the desorption process, e.g., by heating the device via electrical power dissipation. The lower part of Figure 5b shows that when increasing the source–drain voltage temporarily from 30 mV to 10 V, the desorption becomes as fast as the adsorption. The corresponding increase of the power dissipation from 140 nW to 15 mW resets the sensor within 10–20 s. The current‐driven self‐heating of the graphene is estimated to increase the temperature Δ*T* by ≈ 150 K, determined based on Δ*T* = *p*/*r*_0_, where *p* is the dissipated power per area and *r*_0_ = 0.4 kW cm^−2^ K^−1^.^[^ ^48^ ^]^ We have also measured the ethanol sensitivity of the SURMOF/GFET and gauged the ethanol concentration in N_2_ with a commercial alcohol sensor. The sensor—as all commercial sensors—is based on heated metal oxide nanostructures and reacts unspecific to ethanol, methanol, and isopropanol, and many other organic molecules.^[^ ^49^ ^]^ The measurement results are summarized in Figure 5c,d. The SURMOF/GFET sensor can detect ethanol concentrations larger than 0.2 mg L^−1^ (≙100 ppm), and a concentration of 3 mg L^−1^ leads to a 30% change in the conductance. This level of sensitivity is comparable to other GFET sensors responding to alcohol, however, we emphasize that these GFET sensors are not selective to ethanol and do not discriminate against methanol or isopropanol.^[^ ^50^ ^]^ In marked contrast, our SURMOF/GFET has a very high selectivity to ethanol, which makes it a unique sensor that is selective to ethanol with a sensitivity of 100 ppm. Furthermore, it operates at room temperature and is insensitive to humidity. More sophisticated electrical readout schemes would enhance the sensitivity, yet with the simple tracking of the conductance applied here, the SURMOF/GFET sensor is sensitive enough to detect ethanol concentrations that are relevant for breath alcohol determination.^[^ ^51^ ^]^

Now, we attempt to give a microscopic explanation for the observations made, and we begin with the GFET without SURMOF. As transport and Raman data show, the graphene layer underneath the Al_2_O_3_ layer in air and N_2_ is strongly hole doped. This points to negative charges located in or on the oxide layers. Regarding the Al_2_O_3_ layer, it has been reported that the growth by ALD can give rise to negative charge densities on the order of 10^12^ cm^−^^2^, which can be two orders of magnitude higher than what is typically observed for thermally grown SiO_2_.^[^ ^52^ ^]^ Whereas it is unclear how many charges are located within the Al_2_O_3_ and SiO_2_ layers, the alumina surface is certainly terminated with —OH groups after the completion of the ALD process.^[^ ^53^ ^]^ Exposure after growth to ambient air leads to the adsorption of water molecules on the hydrated alumina. Unlike on unhydrated alumina, molecular water is strongly bound to hydrated alumina and can only be removed when heated to 170 °C.^[^ ^54^ ^]^ When measuring the GFET without SURMOF, we have therefore to assume that the Al_2_O_3_ surface is terminated with —OH groups and covered with water molecules, despite purging with N_2_. How those groups get negatively charged is not clear, but probably through an electrochemical electron trapping process by the —OH groups occurring during electrical characterization. This mechanism has been verified by Fourier transform infrared (FTIR) spectroscopy for devices on —OH terminated SiO_2_ surfaces.^[^ ^55^ ^]^ It is reasonable to assume that hydroxyl groups on Al_2_O_3_ are as effective in trapping charges and that the Al_2_O_3_ layer is thin enough to allow the tiny tunneling current that is required to fill the traps. In any case, not all —OH groups of fully hydrated alumina will be charged as this would lead to an excessive surface charge density on the order of >10^15^ cm^−^^2^.^[^ ^56^ ^]^

Next, we need to understand why exposure to alcohol and water reduces the hole‐doping of the GFET. One could think of two reasons: Because of screening of charges or because of charge removal. Simulations show that monohydric alcohol interacts nondissociative with hydrated alumina by an antiparallel alignment of the alcohol polar group to the alumina hydroxyl group.^[^ ^57^ ^]^ Water molecules on hydrated alumina orient in a similar way.^[^ ^56^ ^]^ Both lead to a reduction of surface dipole moments. Moreover, when surface charges are covered by a polarizable medium, the electric field underneath the surface—where graphene is located—will be reduced approximately by 2ε_1_/(ε_1_ + ε_2_), where ε_1_ and ε_2_ are the effective dielectric media below and above the interface, respectively.^[^ ^58^ ^]^ For our devices, the factor could be on the order of 0.5, which would explain the reduction of hole‐doping. In this picture, the screening will disappear and the strong hole‐doping will reappear as soon as a device is purged with N_2_, which experimentally occurs within minutes. The mechanism is kind of similar to the screening induced reduction of hole‐doping observed for graphene devices covered with ionic liquid,^[^ ^59^ ^]^ or metal top gate.^[^ ^60^ ^]^ The physics of diffusive graphene devices can be described by coupled Poisson drift‐diffusion equations^[^ ^61^, ^62^ ^]^ and we have used a finite element partial differential equation solver to approximate the device behavior within computational limitations. We could qualitatively reproduce a reduction of the gate voltage when adding a polarizable dielectric on the top, however, the effect showed to be rather small (see the Experimental Section and Figure S6 in the Supporting Information). Therefore, we also consider charge removal as a possible mechanism. A simple hopping of negative charges from the surface is rather unlikely considering the electron affinities of alcohol (2.1 eV), water (−1.3 eV), and hydroxyl radicals (+1.8 eV). However, a chemical reaction might take place to withdraw charges. From desorption experiments of ethanol on hydrated alumina, it is known that diethyl ether and ethene form and evaporate from the surface when heated above 180 and 230 °C,^[^ ^63^ ^]^ with H_2_ and H_2_O as byproducts. Whether charges at the surface promote reactions and lead to partial charge removal at room temperature remains unclear, but the reduction of hole‐doping could be an indication for it. The recharging in N_2_ back to strong hole‐doping would then have to follow the initial charging mechanism.

One of the possibilities of a chemical reaction that could reduce negative net charges on the alumina surface is chemisorption of alcohol. The reaction of alcohol competes with that of water, thus it depends on the relative concentrations of water versus alcohol near the surface. Bauer et al.^[^ ^64^ ^]^ showed that at high concentration of alcohol (alcohol chemical potential μ_alcohol_ < −1.10 eV) and low concentration of water (water chemical potential μ_W_ < −1.00 eV), alcohol molecules can displace water molecules. As a reference, in ambient conditions, the water chemical potential is μ_W_ (293 K, 1 bar) = 0.57 eV.^[^ ^65^ ^]^ The environment favoring alcohol chemisorption is possible in the experiment when the flow of dry air (nitrogen) is still saturated with alcohol vapor at ambient pressure. The chemisorption of alcohol on alumina causes the alcohol to split into H^+^ and negatively charged alkoxy groups (alkoxy^−^ such as ethoxy C_2_H_5_O^−^). The negatively charged alkoxy^−^ groups, which are weakly bound to the alumina surface, diffuse away from the surface, as was observed in other experiments. Van Tassel and Randall showed that alumina powder carries a positive net charge in 99.9% liquid alcohol caused by desorption of alkoxy^−^ from the surface of alumina.^[^ ^66^ ^]^ The experiment suggests that the chemical bond of alkoxy^−^ to aluminum is the weakest, which makes the charge state of the alumina merged in alcohol positive. On the other hand, chemisorption of water produces H^+^ and OH^−^, OH^−^ just like hydroxylated alumina with OH ends are less likely to be desorbed than alkoxy^−^. This can be seen from the higher binding energy/exothermic chemisorption of water compared to any alcohols.^[^ ^65^ ^]^ As a result, depending on the interplay of the protonation and the removal efficiency of the anions, the surface of alumina can acquire some positive charge density. During the exposure of the GFET with alcohol gas, the positive net charge is caused by the remaining H^+^ at the alumina surface, when alkoxy^−^ is released and drifts away. The quantitative estimation for the electrostatics effect is explained in the next paragraph.

Since we used silicon wafers with oxide thickness of *d*_SiO2_ = 300 nm, we can estimate the charge density on the bottom gate as a function of the bottom gate voltage. If we set bottom gate voltage to *V*_gate_ = 60 V = *E*·*d*_SiO2_, then the charge density is σ = ε_SiO2_·ε_0_·*E* ≅ 4.3 × 10^12^ cm^−2^, where ε_SiO2_ is the permittivity of SiO_2_ with a dielectric constant of 3.9 times the vacuum dielectric constant ε_0_ = 8.85 × 10^12^ F m^−1^, and *E* is the electric field. The effect of *V*_gate_ = 60 V can be replaced by the same value of surface charge density σ ≅ 4.3 × 10^12^ cm^−2^ at “top gate” or alumina surface if the bottom gate is at 0 V (Figure S7, Supporting Information). In the experiment, we observed roughly 60 V left shift of the Dirac voltage when alcohol gas was present. For α‐alumina, we approximate the full coverage due to chemisorption of alcohol on flat alumina surface to be one pair of alkoxy^−^ and H^+^ adsorption sites per 26.4 Å^2^, or 3.78 × 10^14^ cm^−2^ adsorption sites (Figure S8, Supporting Information). Therefore, we estimate that 1.2% protonated surface area of a perfectly flat alumina surface can create 60 V left shift provided the same amount of alkoxy^−^ is removed. In the gas phase, the order of the acidity is isopropanol > ethanol > methanol > water. During chemisorption of alcohol in alumina, alcohol produces H^+^ and alkoxy^−^. The alkoxy^−^ has a lower binding energy than H^+^, which has been observed in experiments. However, methanol, as the smallest alcohol, has the highest possible chemisorption site density compared to the other alcohols. This agrees with the experimental observations in Figure S4 in the Supporting Information, where methanol has the highest left shift at more than 50 V followed by ethanol and isopropanol at about 30 V, and finally a very nominal left shift with water. The n‐doping effect of water is an additional signature that water molecules do not make a direct contact with graphene layer since water is a p‐dopant when directly interacting with graphene.^[^ ^44^ ^]^ The recharging in N_2_ back to strong hole‐doping would then have to follow the initial charging mechanism.

After the SURMOF has grown on the surface, fewer hydroxyl groups are left because of the chemical reaction with the copper acetate that anchors the SURMOF to the alumina. A very small number of charged OH groups is sufficient to explain the close to zero doping and the high mobility that is observed after the SURMOF growth procedure. However, zero doping could also be a result of compensation of the remaining negatively charged —OH groups by positive charges. Unfortunately, the initial stages of the SURMOF growth remain elusive. In the ideal structure, there are 1.5 Cu‐paddle‐wheel‐metal‐nodes per nm^2^, nominally bound to one OH‐group per metal node. The anchoring of Cu in the first layer formation would affect a smaller fraction of the original —OH group density and the total charge at the alumina surface. On the other hand, the appearance of positive charges during synthesis has not been established, although the existence of charged defects in related SURMOFs structures has been reported recently.^[^ ^67^, ^68^ ^]^ In the first layer, the BDC‐ligands can only attach to 1 out of 4 sites to neutralize two Cu^2+^ atoms on the Cu‐BDC paddle wheel secondary building unit (SBU) of the SURMOF caused by the steric (molecular size) restriction. The BDC‐ligands can attach only from the top part of Cu paddle wheel SBU. Therefore, a high concentration of positive charge caused by the excess of positive Cu^2+^ ions may form on the interface between SURMOF and alumina. This positive charge layer may shift the tendency of ethanol from positive charge donor (Dirac voltage left shift) to negative charge donor (Dirac voltage right sift) to the system. This interface may react with ethanol, which has higher acidity than methanol in gas phase and thereby reduce the excess of the positive charge in the alumina and SURMOF interface. Addition of negative and/or removal of positive charges could lead to hole‐doping as observed for ethanol. At this point, we cannot provide a complete microscopic model to fully explain the different behavior of the alcohols on the alumina–SURMOF interface.

Our calculations with the finite element method (FEM) shows that the effect from dipole interactions or dielectric polarization alone cannot explain the large Dirac voltage shift (>10 V). Here, we also determine that gas–graphene direct interactions are very unlikely, water is supposed to be a p‐dopant but we observed water as an n‐dopant in our sample without SURMOF.^[^ ^44^ ^]^ This narrows down the main mechanism to the long‐range (3–5 nm) electrostatic effect as a result of chemisorption of gases with alumina and interface between alumina and SURMOF. We believe that future in situ/in operando FTIR spectroscopy, targeted toward detection of the relevant molecular species and processes at the interface, shall provide the necessary information to fully resolve the sensing mechanism.

In summary, we have demonstrated a novel sensing principle by interfacing a MOF film with a graphene transistor. Given the countless variations of MOF films and the possibilities to chemically engineer the interface between MOF and GFET, we envision the emergence of a whole new class of MOF/GFET sensors with tailored selectivity and sensitivity.

## 3 Experimental Section

### SURMOF Synthesis

The SURMOF layer was grown by the layer‐by‐layer (LbL) synthesis as described by Liu et al.^[^ ^33^ ^]^ First, the surface of the GFET devices was activated in a UV ozone cleaner (Ossila, Sheffield, UK) for 1 min to maximize the number of functional OH groups at the Al_2_O_3_ surface. Afterward, the devices were put immediately into a 1 × 10^−3^ m of copper(ii) acetate (Cu_2_(OAc)_4_(H_2_O)_2_) ethanol solution before the synthesis. Then, the devices were placed on the sample holder and subsequently sprayed with 1 × 10^−3^ m Cu(OAc)_2_ ethanolic solution for 15 s and with a 0.2 × 10^−3^ m 1,4‐benzene dicarboxylic acid (BDC) ethanol solution for 25 s at room temperature. Between both the steps, the sample was thoroughly rinsed with pure ethanol to remove undercoordinated metal‐nodes or organic linker molecules. This procedure was repeated (in total) 35 times to grow a nominally 100 nm thick layer.

### Device Fabrication

Monolayer graphene on 300 nm thick thermal SiO_2_ on p/B‐doped 〈100〉 Si 1–10 Ω cm, 525 µm thick (from Graphenea) was used for the GFET fabrication. The SURMOF/GFET fabrication required five electron‐beam lithography steps prior SURMOF growth (see process flow in Figure 1). For all the steps, poly(methyl methacrylate) (PMMA) 950k resist (Allresist) diluted in anisole was used and prebaked at 150 °C on a hot plate for 3 min. The e‐beam exposed areas were developed in a solution of methyl isobutyl ketone/isopropanol (MIBK/IPA) for 30 s, rinsed with IPA, and dried in a nitrogen stream giving the required patterned structure. In the first step, markers were defined. PMMA A4.5 (4.5% PMMA in anisole) was coated at 5000 rpm for 60 s, e‐beam patterned, and developed. 50 nm tungsten was deposited by sputtering (Bestec, 300 W_DC_, 60 s) and subsequently lifted‐off in acetone. Next, graphene strips of dimensions 5 µm × 100 µm were defined and perforated with holes (230 nm diameter, 640 nm lattice spacing, in total 2×(75 × 5)) by spin coating PMMA A4.5 at 4000 rpm for 60 s, e‐beam patterning, cold development at 0 °C, and by etching of the graphene around the PMMA protected strips and in the holes via oxygen plasma in a reactive ion etcher (RIE Oxford Plasmalab 80 plus, 15 sccm O_2_, 60 mTorr, 30 W for 75 s) leaving an unperforated area in the middle of 5 µm × 5 µm. In the next step, the source–drain electrodes and the back gate contacts were defined. A scratch through the SiO_2_ was made to contact the Si gate. After spin‐coating PMMA A4.5 at 6000 rpm for 60 s, e‐beam exposure, and development, 3 nm Cr and 42 nm Pd were deposited by sputtering (100 W, RF, 30 s, and 70 W, DC, 45 s, respectively). On this fabricated GFET device, 5 nm of aluminum oxide was grown using TMA and ozone at 150 °C by thermal atomic layer deposition (TMA pulse time = 0.1 s at 150 sccm flow rate, *T* = 25 °C, purge time = 6 s, oxygen pulse time = 0.1 s at 100 sccm flow rate, purge time = 6 s, power = 70%, 75 cycles). The Al_2_O_3_ layer was locally removed for electrical contacting by first spin‐coating PMMA A8 (8% PMMA in anisole) at 6000 rpm, e‐beam patterning, and development. Then, the aluminum oxide was etched in the RIE (40 sccm Ar, 10 sccm CHF_3_, 200 W, 15 mTorr, 70 s). In the fifth and final step, the area for the SURMOF growth on top of the devices was defined by spin coating PMMA A4.5 at 5000 rpm, e‐beam patterning, and development.

### XRD

X‐ray diffraction measurements were carried out in an out‐of‐plane geometry using a Bruker D8‐Advance diffractometer equipped with a position‐sensitive LynxEye detector in θ–2θ geometry. A Cu‐anode with a wavelength of λ = 0.154 nm was used. The samples were investigated with an angle increment of 0.02° and a scan speed of 4 s per step.

### SEM/EDX

Scanning electron microscopy images were taken with a Zeiss Ultra plus SEM at 10 keV beam energy, at 45° tilt angle, and inlens detection. Energy‐dispersive X‐ray spectra were recorded with a Zeiss LEO 1530 SEM and an Oxford instruments X‐maxN detector at 4 keV beam energy, and analyzed with AZtec software from Oxford instruments.

### Raman

Spectra of CVD‐graphene/300‐nm‐SiO_2_/Si sample were measured before and after Al_2_O_3_ and SURMOF growth, under ambient conditions with a Renishaw inVia Raman microscope at 532 nm excitation wavelength, 3 mW power, 60 s integration time, and 20× magnification. SURMOF/5‐nm‐Al_2_O_3_/graphene/300‐nm‐SiO_2_/Si reference sample was measured under ambient conditions after exposure to ethanol (99.96% purity, VWR chemicals), isopropanol (>99.8% purity, Carl Roth), and methanol (99.9% purity, Merck) vapor. Before alcohol vapor exposure, the samples were vacuum annealed at 150 °C. The data were acquired at 532 nm excitation wavelength, 0.6 mW (after alcohol exposure) and 3 mW (after vacuum annealing), 60 s integration time, 20× magnification.

### Electrical Transport and Sensing Measurements

The GFETs and SURMOF/GFTEs were mounted to a ceramic chip carrier, wire bonded, and with the package mounted to a cavity of volume ≈1 cm^3^. The cavity has an inlet and outlet as part of a gas line system as described by Ganzhorn et al.^[^ ^36^ ^]^ A four‐way valve enables instantaneous switching between gases, and the conditions were controlled by pressure gauges and flow meters (dosing valves). All measurements were carried out at flow rates of 0.5 L min^−1^ and a static gas pressure of 0.25 bar above atmospheric pressure. The dynamic pressure (<10^−4^ bar) was negligible. The gas in the cavity was exchanged within 0.1 s, setting the time resolution of the measurements. In the downstream, a humidity sensor (Bosch BMP280) and an alcohol sensor (NCD MQ‐3) were mounted in an additional cavity and monitored with a Raspberry Pi. For exposure to alcohol and water, the liquids were filled into a bubbler and dry nitrogen was passed through at a flow rate that yields the carrier gas saturated with the corresponding liquid, as monitored by the downstream sensors. If not stated otherwise, the initial starting condition was flowing dry nitrogen, and measurements were taken ≈15 min after changes of conditions. Electrical transport measurements were carried out with an Agilent 4155C semiconductor parameter analyzer with TRIAX cabling, and back gate sweeps were conducted at source–drain voltages of 0.01, 0.02, and 0.03 V. Time‐dependent measurements were done at zero back gate voltage and 1 s time intervals. All experiments were carried out at room temperature.

### Simulation

Proof‐of‐principle simulations of the graphene carrier concentration and Dirac voltage as a function of back‐gate voltage and surface charge density were conducted using a finite element partial differential equation solver (FlexPDE 6). The coupled Poisson drift‐diffusion equations were solved for the electrostatic potential and the quasi‐Fermi potentials^[^ ^69^ ^]^ by solving iteratively the Laplace equation and the drift‐diffusion equations.^[^ ^61^, ^62^ ^]^ The surface charge density was imposed by a natural boundary condition. The code can be found at github.com/krupke‐group.

The selectivity of the gases was simulated by SURMOF pores at the DFT level for physisorption and diffusion barrier energies, and classical Grand Canonical Monte Carlo (GCMC) simulations for the uptake of gases at ambient temperature and pressure. The diffusion barrier energy in SURMOF was calculated with the nudged elastic band (NEB) method^[^ ^70^, ^71^ ^]^ using the Vienna Ab initio Simulation Package (VASP 5.4.4). For details, refer to Figure S9 in the Supporting Information. The physisorption energy and diffusion energy barrier of the alcohols is shown in **Table** **1**. The vdW‐optB86b functional^[^ ^72^ ^]^ that already incorporates van der Waals corrections in the DFT simulation was used.

**Table 1:** Physisorption energy and diffusion energy barrier of alcohol in Cu‐BDC SURMOF

Energy [kJ mol^−1^]	Methanol	Ethanol	Isopropanol
Physisorption energy	−56.0	−54.4	−78.5
Diffusion energy barrier	16.4	19.5	42.8

**Figure 6:**
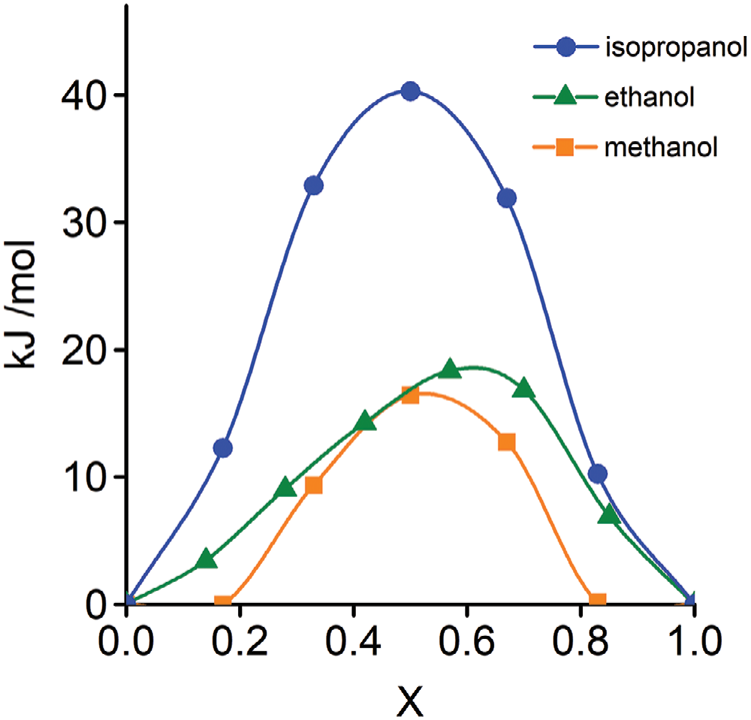
Diffusion energy barrier of the alcohols in SURMOF (Cu‐BDC) calculated by the nudged elastic band (NEB) method in density functional theory (DFT). *X* is the reaction coordinate along the pore axis and the maximum barrier energy is close to the pore window *X* ≈ 0.5. The energy barriers are: 16.4 kJ mol^−1^ for methanol (yellow squares), 19.5 kJ mol^−1^ for ethanol (green triangles), and 42.8 kJ mol^−1^ for isopropanol (blue circles).

It is observed that the physisorption energy of these alcohol are between −56 and −78.5 kJ mol^−1^. Isopropanol was the highest (−78.5 kJ mol^−1^) per molecule because it had the largest surface areas for van der Waals interactions with the pore surface. The small difference in physisorption energy between methanol (−56 kJ mol^−1^) and ethanol (−54.4 kJ mol^−1^) was caused by the possible closest distance of the OH head interacting with Cu–COO on paddle wheel SBU and molecular size/surface. The diffusion energy barrier of isopropanol at 42.8 kJ mol^−1^ was more than twice that of methanol at 16.4 kJ mol^−1^ and ethanol at 19.5 kJ mol^−1^ (**Figure** **6**). At the DFT level, all alcohols were predicted to be able to be adsorbed by the SURMOF. However, isopropanol may not diffuse deeper than 35 layers of SURMOF deposited by LbL method. These results agree with Raman measurements, which did not detect isopropanol on the alumina surface after SURMOF deposition.

Results of GCMC simulations of saturated alcohols at atmospheric pressure (water 3.17%, methanol 17%, ethanol 8%, isopropanol 6%) in N_2_ in the SURMOF pores and experimental values are shown in **Table** **2**.

**Table 2:** Adsorption of alcohol gases at their saturated pressure in nitrogen gas at 1 atm, 298 K

Uptakes	Per unit cell			Ratio		
Gas type	Methanol	Ethanol	Isopropanol	Methanol	Ethanol	Isopropanol
Experiment	0.5	0.3	0.2	2.5	1.6	1
Sim. pure alcohol	3.8	2.5	1.5	2.5	1.7	1
Sim. alcohol + N_2_	3.5	1.7	1.3	2.6	1.3	1

The uptake for all alcohols in Table 2 was simulated at room temperature 298 K and 1 atm total pressure (alcohol + nitrogen gases). Simulation data were also included for pure alcohol at their saturated partial pressure values (0.17, 0.08, and 0.06 atm for methanol, ethanol, and isopropanol, respectively). All alcohols can be absorbed in SURMOF. Water had a much higher uptake, which was 9 per unit cell in GCMC, water presented in the SURMOF Cu‐BDC structures through hydrogen bonding between 2D layers of Cu‐paddle wheel SBU. The ratio of the uptakes between methanol:ethanol:isopropanol = 2.6:1.3:1 from the simulations was very close to the experiments 2.5:1.6:1. The experimentally determined adsorption of the alcohols per unit cell was about 15% of the theoretical/simulation uptake values. This was expected since the porosity of SURMOF 2D structure is not as high as typical 3D MOF with much higher accessible pore surface. Tower‐like structures may also form on Cu‐BDC instead of homogenous 35 layers SURMOF and reduce the total accessible surface area of the pores. From these calculations, the cause of the selectivity was excluded as exclusively from the physisorption or diffusion barrier of the gases alone since both methanol and ethanol are easily adsorbed and diffuse at ambient temperature and pressure.

## Conflict of Interest

The authors declare no conflict of interest.

## Author Contributions

The experiments were conceived and designed by R.K., L.H., C.W., and W.W. Devices were fabricated and characterized by S.K., S.D., K.M., and A.C. with input from R.K., L.H., and D.P. Simulations were performed by R.K., Y.P., S.H., and M.R. The manuscript was written by R.K., S.K., Y.P., S.H., and A.F., with input from all coauthors.

## Supporting information

Supporting Information

## Data Availability

Research data are not shared.
