# Supplementary material for: Sensing Molecules with Metal–Organic Framework Functionalized Graphene Transistors
Source: Adv Mater. 2021 Sep 8;33(43):2103316. doi: 10.1002/adma.202103316 (PMC11469265; doi:10.1002/adma.202103316)
Supplement: Supplementary file 1 — Supporting Information [file ADMA-33-2103316-s001.pdf]

© 2021 Wiley-VCH GmbH

ADVANCED MATERIALS

Supporting Information

for *Adv. Mater.,* DOI: 10.1002/adma.202103316

Sensing Molecules with Metal–Organic Framework Functionalized Graphene Transistors

Sandeep Kumar, Yohanes Pramudya, Kai Müller, Abhinav Chandresh, Simone Dehm, Shahriar Heidrich, Artem Fediai, Devang Parmar, Delwin Perera, Manuel Rommel, Lars Heinke, Wolfgang Wenzel, Christof Wöll, and Ralph Krupke**

Sensing molecules with metal-organic framework functionalized graphene transistors

Sandeep Kumar[1,4], Yohanes Pramudya[1], Kai Müller[3], Abhinav Chandresh[3], Simone Dehm,[1] Shahriar Heidrich[1], Artem Fediai[1], Devang Parmar[2], Delwin Perera[4], Manuel Rommel[1], Lars Heinke[3], Wolfgang Wenzel[1], Christof Wöll[3], Ralph Krupke[1,2,4]

[1] Institute of Nanotechnology, Karlsruhe Institute of Technology, 76021 Karlsruhe, Germany

[2] Institute of Quantum Materials and Technologies, Karlsruhe Institute of Technology, 76021 Karlsruhe, Germany

[3] Institute of Functional Interfaces, Karlsruhe Institute of Technology, 76021 Karlsruhe, Germany

[4] Institute of Materials Science, Technische Universität Darmstadt, 64287 Darmstadt, Germany

Supporting Information

- Energy-dispersive X-ray (EDX) data of samples before and after SURMOF growth (Figure S1)
- X-ray diffraction (XRD) analysis of the SURMOF layer (Figure S2)
- Scanning electron microscopy (SEM) imaging of the SURMOF layer (Figure S3)
- Response of the GFET without SURMOF to alcohol and water molecules (Figure S4)
- Alcohol adsorption and desorption measurements (Figure S5)
- Finite element simulation (Figure S6)
- Sensing principle (Figure S7)
- Modelling alcohol chemisorption (Figure S8)
- Illustration of the Nudged Elastic Band (NEB) method (Figures S9)

Energy-dispersive X-ray (EDX) data of samples before and after SURMOF growth

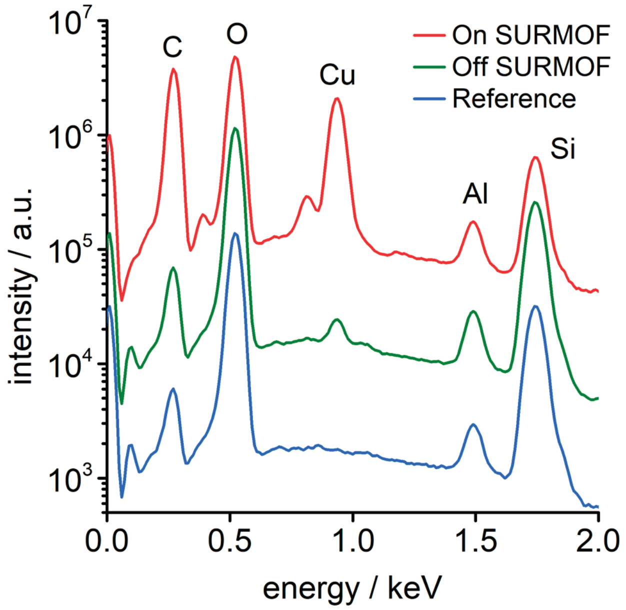

Figure S1: Energy-dispersive X-ray (EDX) spectra for evaluating the integrity of the ALD-grown Al_2O_3 layer. The reference is a 5nm-Al_2O_3/graphene/300nm-SiO_2/Si sample, on which a nominally 100 nm thick SURMOF layer was grown. The primary beam energy was 4 keV, sufficient to probe the Al_2O_3 layer underneath the top layer (On SURMOF). The upper two curves are shifted for clarity. Residues of copper from the SURMOF are detectable in lifted-off regions (Off SURMOF). See also Figure S3.

The energy-dispersive X-ray data shows that the ALD-grown Al_2O_3 layer remains intact after the SURMOF synthesis since the Al peak is only slightly attenuated underneath the SURMOF (On SURMOF). The attenuation is due to the SURMOF layer on top and is seen more clearly when comparing the Si peak of the underlying substrate. The SURMOF itself gives rise to the large Cu peak and the enhanced C peak. The small Cu peak in the lifted-off regions (Off SURMOF) is coming from SURMOF residues which can also be seen in the SEM images in Figure S3.

X-ray diffraction (XRD) analysis of the SURMOF layer

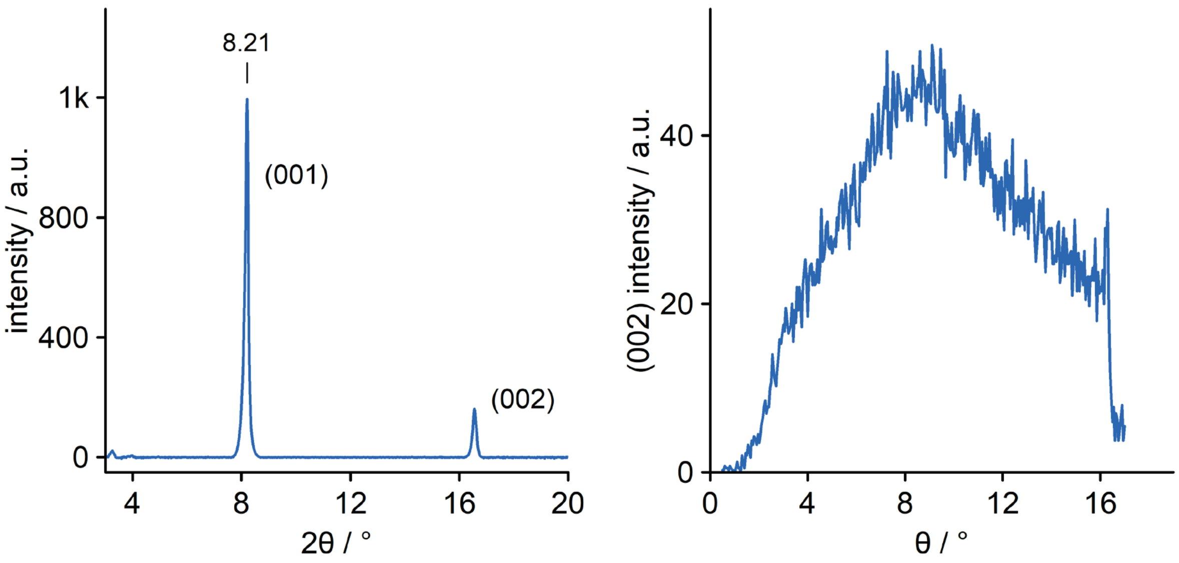

Figure S2: X-ray diffraction (XRD) analysis of the $Cu_2(BDC)_2$-SURMOF-2/5nm-Al_2O_3/graphene/300-nmSiO_2/Si sample. (a) Out-of-plane XRD data, (b) Rocking curve of (002) reflex. X-ray source: $CuK_{\alpha1}$ line, sample size: 5mmx5mm.

Scanning electron microscopy (SEM) imaging of the SURMOF layer

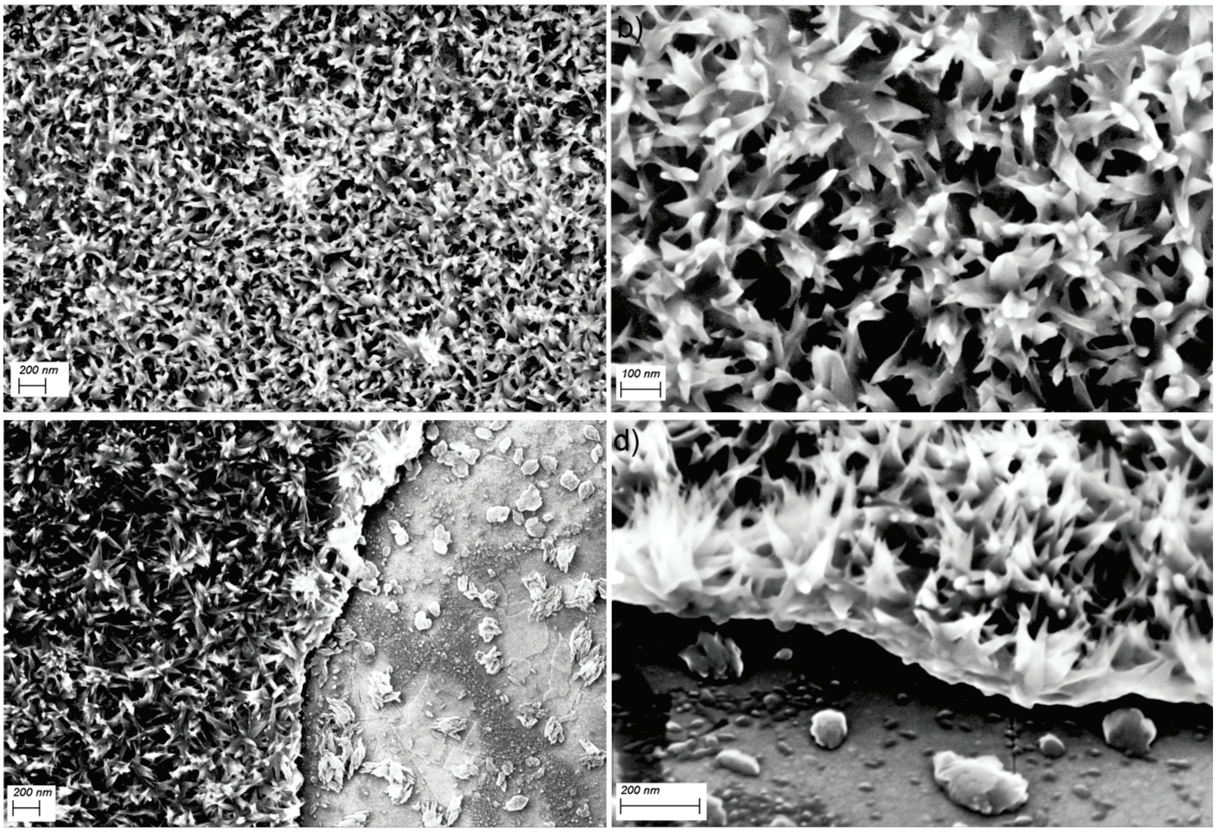

Figure S3: Scanning electron microscopy (SEM) images of a SURMOF/5nm-Al_2O_3/graphene/300-nmSiO_2/Si sample, recorded at 10 keV and with in-lens detection. (a-c) measured at normal incidence, (d) under 45° angle from the front. (c-d) show the edge of the SURMOF after partial lift-off.

The scanning electron microscopy (SEM) images show the morphology of a nominally 100 nm thick SURMOF layer from a top view (Figure S3a-c) and under an angle of 45° (Figure S3d). In Figure 3c-d we look at an edge of the SURMOF layer that formed by lifting off the PMMA mask which defined the area of SURMOF growth on the Al_2O_3 surface. Figure S3d shows an edge segment that is bent upwards and gives a view onto the bottom of the SURMOF layer. Within the spatial resolution of the SEM, the bottom surface appears continuous, whereas the upper surface is very rough with small MOF crystallites pointing in various directions.

Response of the GFET without SURMOF to alcohol and water molecules

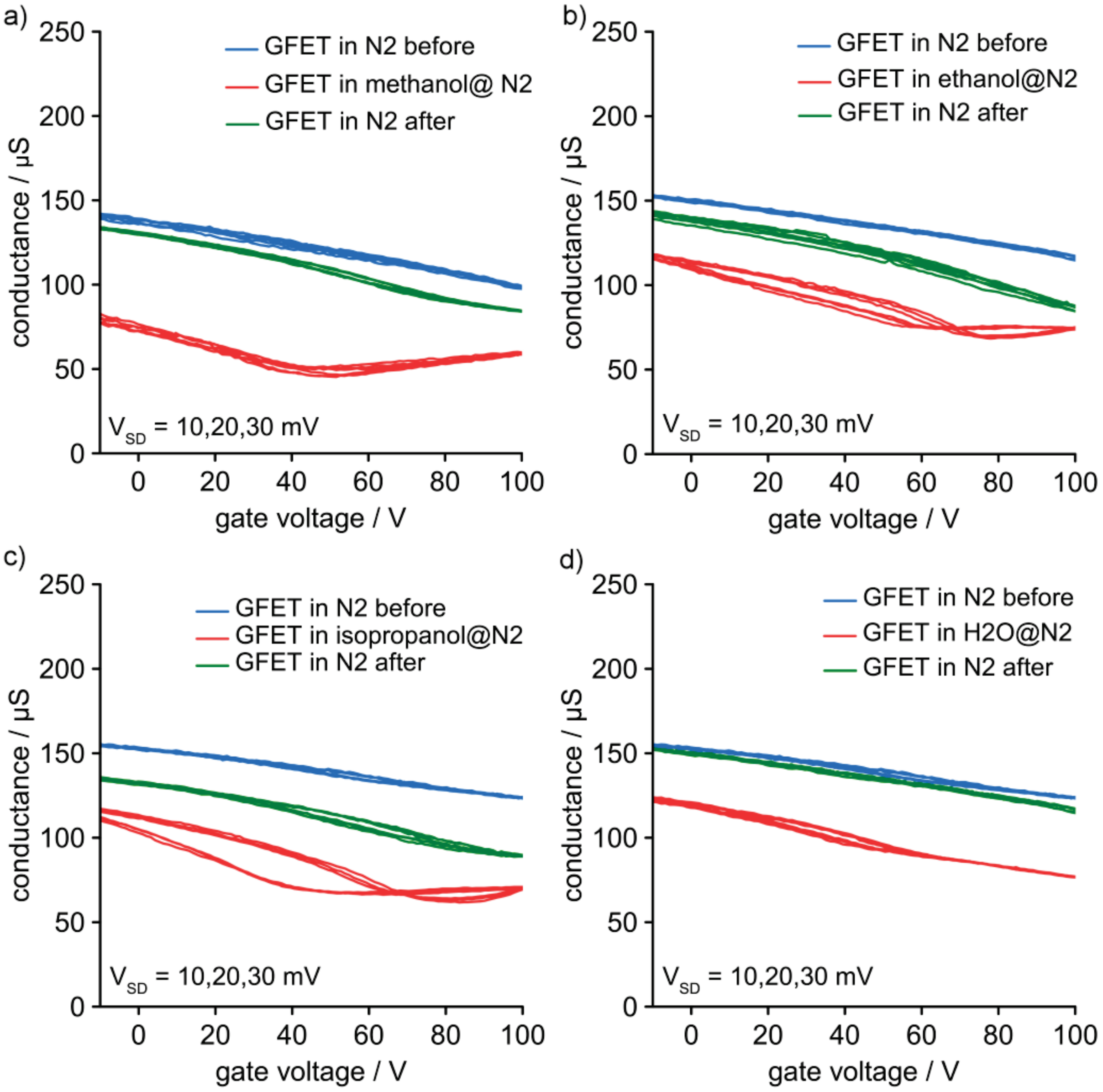

Figure S4: Response of the GFET without SURMOF to methanol- (a), ethanol- (b), isopropanol- (c), and water molecules in the N_2 stream. The conductance versus gate-voltage is shown for the GFET in N_2 before and after the exposure. Source-drain voltages are indicated.

Alcohol adsorption and desorption measurements

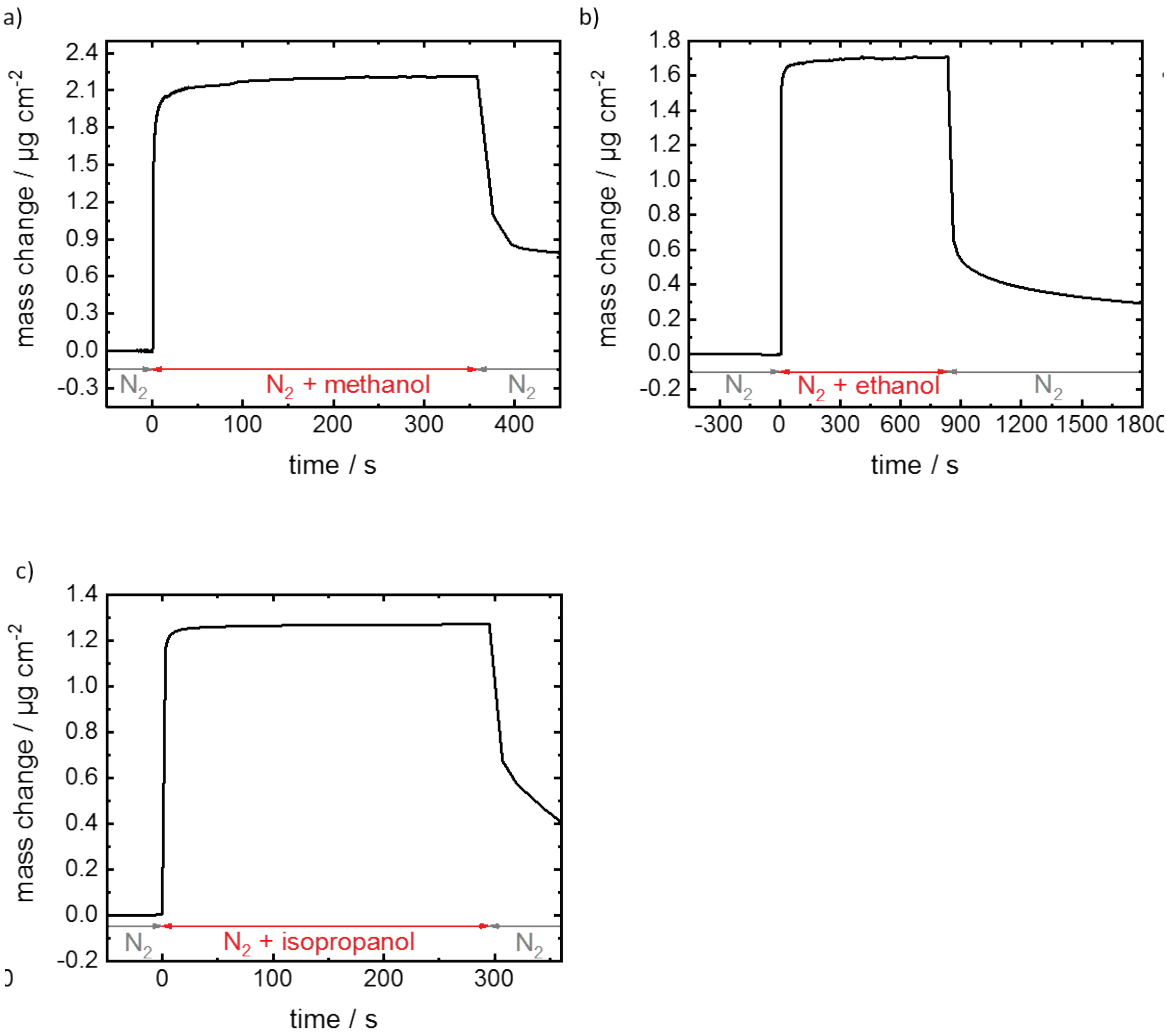

Figure S5: Vapor uptake by the MOF film grown Au@QCM-sensors. The vapors are a) methanol, b) ethanol and c) isopropanol. Initially the samples are activated in an atmosphere of pure nitrogen, then the gas flow is instantaneously enriched with the vapor of the guest molecules with a vapor pressure close to saturation. After reaching equilibrium, the gas atmosphere is switched back to pure nitrogen. The gas flow rate is 100 ml per min. The temperature is 25°C.

Finite element simulation

a) b)

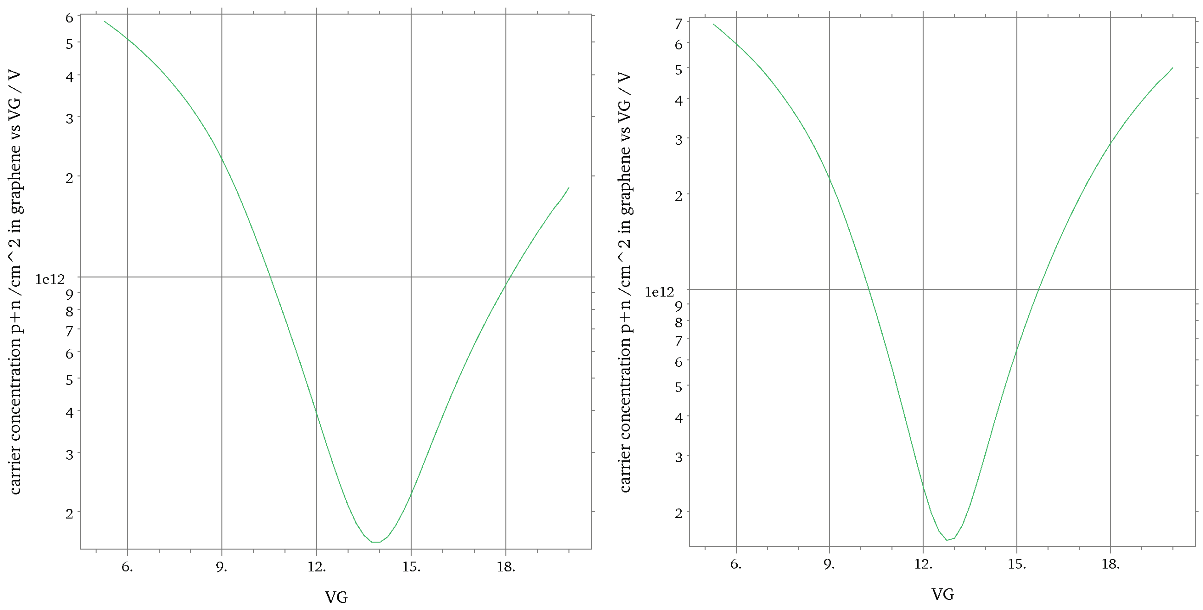

Figure S6: Simulated charge carrier concentration p+n in graphene vs back-gate voltage V_G at 10 mV source-drain bias. Graphene was modelled as a 50 nm x 50 nm x 1 nm layer with a 30 nm back-gate dielectric (eps_SiO_2), a 3 nm top dielectric (eps_Al_2O_3), and a surface charge density of -10^{13} cm^{-2}. (a) Simulation with a 30 nm layer (eps=1) on top, and (b) with a 7 nm (eps=10^3) and a 13 nm (eps=1) layer on top. The data was taken from the center of the graphene layer. The high-k dielectric layer on top of the negative surface charge reduces the Dirac voltage and narrows the Dirac cone.

Sensing principle

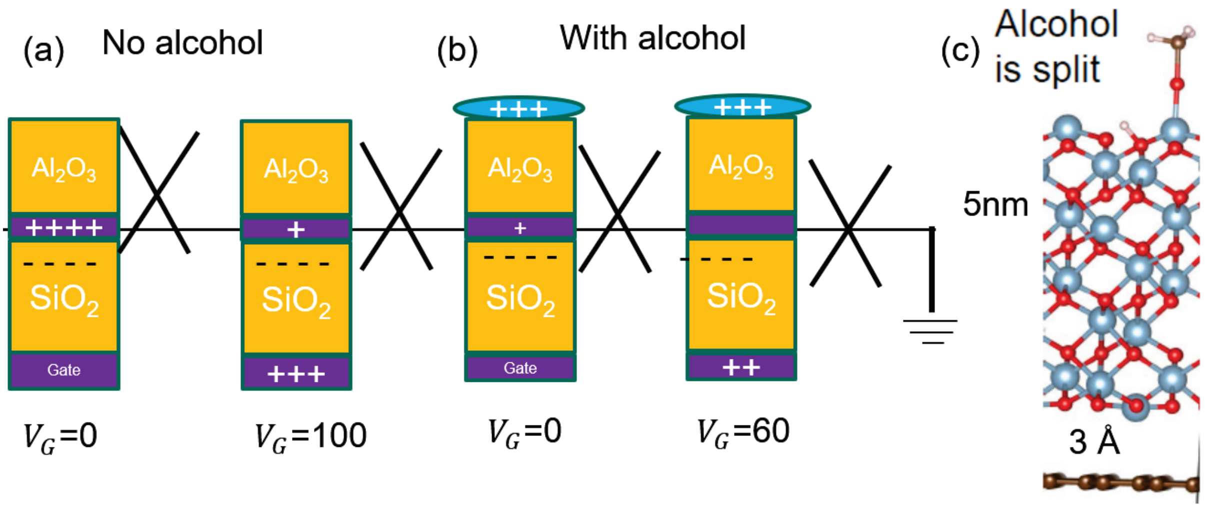

Figure S7: Sensing principle of the graphene FET with Alumina covered gate. (a) Without alcohol, the graphene is highly hole-doped and the Dirac voltage is located beyond 100 V gate voltage. (b) With alcohol the Dirac voltage is located at 40-60 V. (c) Positive charge accumulated at alumina acts as the second gate which electrostatically doped graphene with electrons. As a result, the charge neutrality point is observed already at a voltage of about 60 V as compared to >100 V for the system not exposed to alcohol.

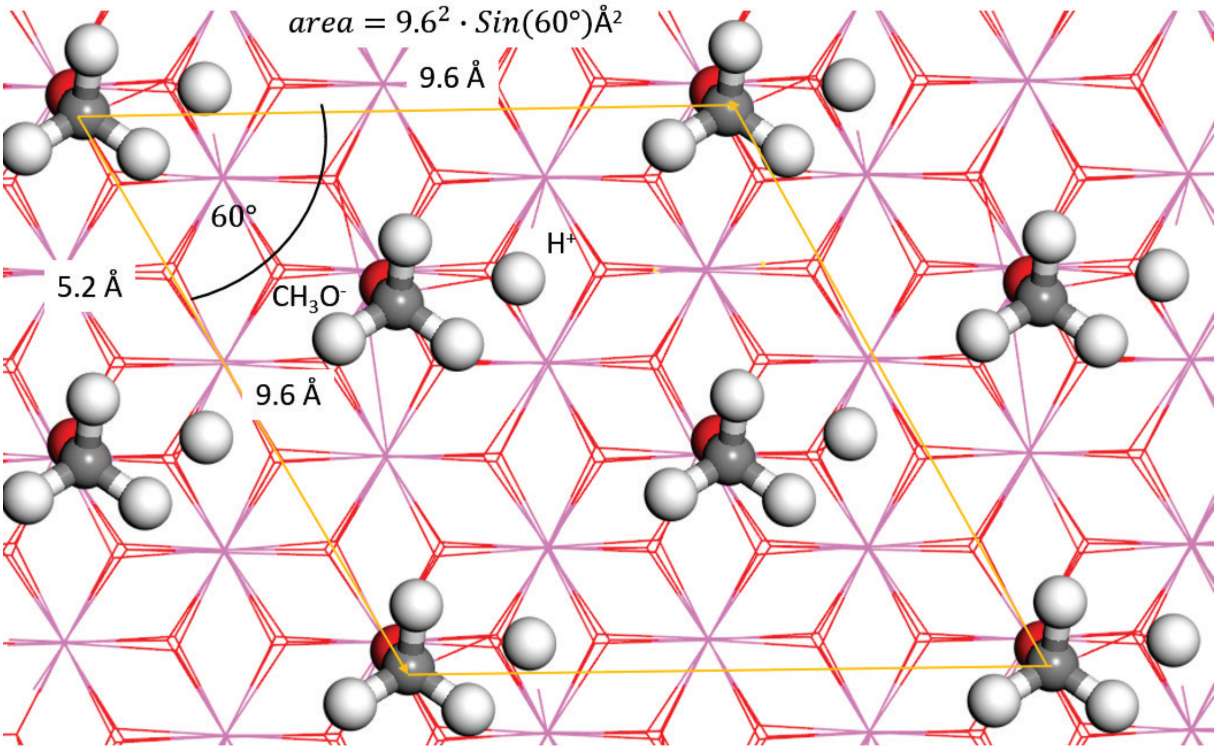

Figure S8: Illustration of the alcohol (methanol) chemisorption on top of α-alumina flat surface. Methanol is split into methoxy$^-$ and H^+ density. The methoxy bonds to alumium atom and hydrogen bonds to oxygen. The rhombus perimeter (yellow line) with the side length 9.6 Å and angle of 60 ° contains 3 sorption sites.

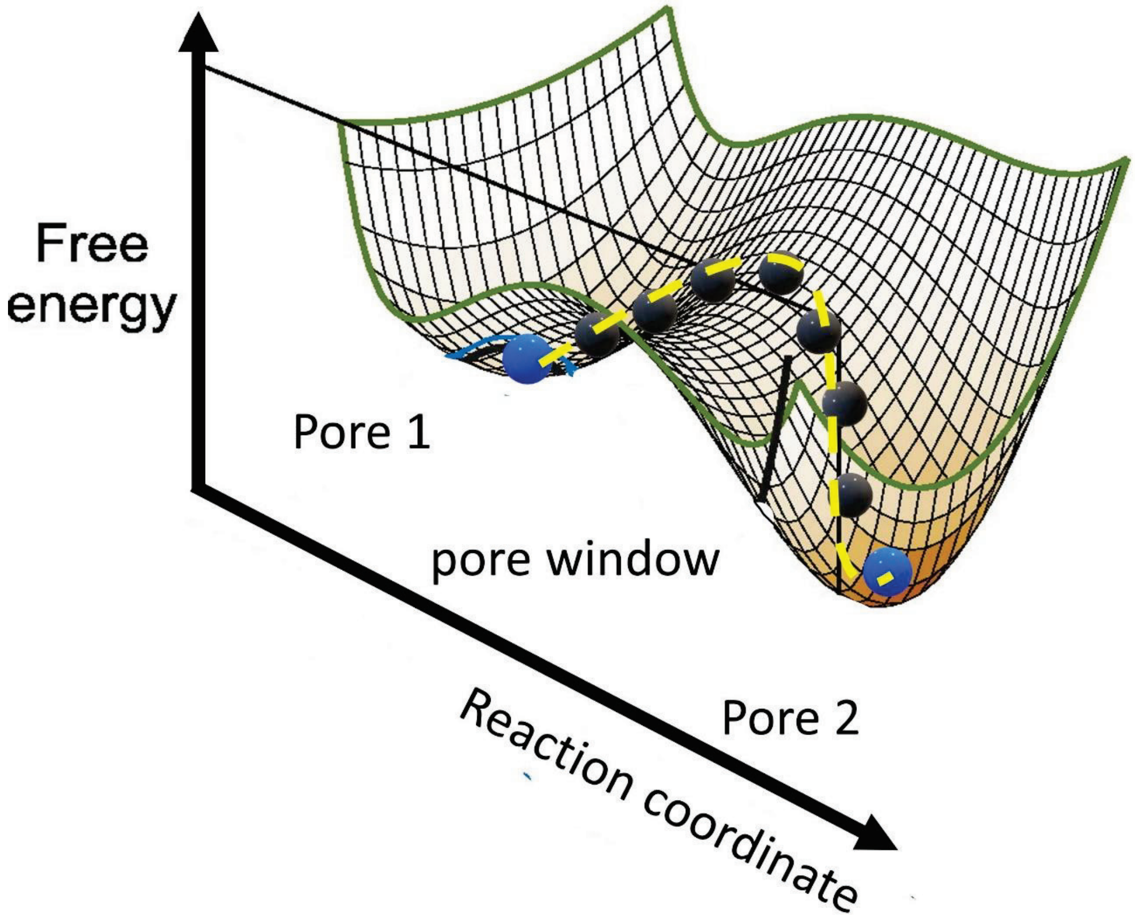

Figure S9: Illustration of the Nudged Elastic Band (NEB) method. First alcohol molecule (blue ball) is placed in pore 1, and the second alcohol molecule (blue ball) is placed in pore 2 inside the MOF. Each of them is geometrically optimized and will reach the lowest energy in the pore 1 and pore 2. We create images of alcohol molecules (7 grey balls) that are connected by spring (yellow dashed line) and form a chain of molecules connecting the alcohol (blue balls) in pore 1 and pore 2. During the NEB process, both ends are not moving because they are already at the minimum energy. The molecule images (grey balls) are minimized simultaneously and will position themselves on their local minima following the contour of the energy surface but restricted by the chain/forces between the images. The molecule images roll down and one of them will occupy the saddle point (in the pore window) by the climbing image algorithm in the reaction path/coordinate. When the NEB process reaches the energy (10^{-6} eV) and force (0.05 eVÅ^{-1}) tolerance, the lowest energy barrier from pore 1 to pore 2 is found.
